# Reverse zoonotic tuberculosis transmission from an emerging Uganda I strain between pastoralists and cattle in South-Eastern Nigeria

**DOI:** 10.1186/s12917-019-2185-1

**Published:** 2019-12-04

**Authors:** Hezekiah Kehinde Adesokan, Victor Oluwatoyin Akinseye, Elizabeth Maria Streicher, Paul Van Helden, Rob Mark Warren, Simeon Idowu Cadmus

**Affiliations:** 1 Department of Veterinary Public Health and Preventive Medicine, University of Ibadan, Ibadan, Nigeria; 2 DST-NRF Centre of Excellence for Biomedical Tuberculosis Research/SAMRC Centre for Tuberculosis Research, Division of Molecular Biology and Human Genetics, Faculty of Medicine and Health Sciences, Stellenbosch University, Tygerberg, South Africa; 3 Center for Control and Prevention of Zoonoses, University of Ibadan, Ibadan, Nigeria

**Keywords:** Zoonoses, Molecular tools, Epidemiology, *Mycobacterium tuberculosis* complex, Public health

## Abstract

**Background:**

Tuberculosis remains a major public health challenge globally with increasing risks for inter-transmission between pastoralists and cattle in Nigeria. This study was aimed at using molecular tools to establish zoonotic transmission of tuberculosis between pastoralists and their cattle in Ebonyi State, Nigeria. Sputum (*n* = 149) and milk (*n* = 144) samples from pastoralists and cattle, respectively were screened on the assumption of subclinical infections considering unguarded human-livestock interactions. Isolates obtained were analysed using deletion typing, spoligotyping and 24-Mycobacterial Interspersed Repetitive Unit-Variable Number Tandem Repeats (MIRU-VNTR).

**Results:**

Fifty-four MTC were confirmed by deletion typing and were differentiated accordingly (*M. tuberculosis*: pastoralists =42, cattle = 2; *M. bovis*: pastoralists =1; *M. africanum*: pastoralists =9). Spoligotyping indicated 59.2% Uganda I/SIT46 (pastoralists =28; cattle = 1), 16.3% Latin American Mediterranean/SIT61 (pastoralists =8), 2.0% T/SIT53 (pastoralists =1) strains of *M. tuberculosis* and new strains of *M. bovis* and *M. africanum*. The 24-MIRU-VNTR of selected predominant cluster isolates shared by cattle and pastoralists (Uganda I/SIT46: pastoralists =9; cattle = 1) showed the same number of copies at each of the repetitive loci.

**Conclusions:**

*Mycobacterium bovis* was confirmed in humans and a reverse zoonotic tuberculosis transmission from an emerging Uganda I *M. tuberculosis* strain between pastoralists and cattle in Nigeria evidenced by MIRU-VNTR. Using molecular tools will help mitigate disease burden through informed epidemiological insights.

## Background

Tuberculosis (TB) remains one of the most important threats to human and animal health. Currently, TB, particularly bovine tuberculosis (BTB) in humans also known as zoonotic TB is gaining awareness as a potentially important problem in developing countries, where no or only limited control measures are applied [1]. The United Nations Sustainable Development Goal 3 includes a target for ending the global TB epidemic and calls for diagnosis and treatment of every person with TB and that patients with zoonotic TB must be included. The intrinsic resistance of *Mycobacterium bovis*, the primary cause of BTB to pyrazinamide [2] and the fact that *M. bovis* patients are twice more likely to die during treatment [3] have made zoonotic TB in humans of great concern. The practice of humans and animals sharing the same micro-environment and dwelling premises has potentiated the disease [4]. Cough spray from infected cattle may be inhaled by occupationally exposed individuals resulting in typical pulmonary TB [5].

Within Africa, available evidence suggests that *M. bovis* causes approximately 2.8% of pulmonary TB cases, for a crude incidence of seven cases per 100,000 population [6]. In 2017, estimated incidence and mortality from *M. bovis* TB in Africa were 70,300 and 9270, respectively [7], although this is likely to be a considerable underestimate, given the lack of definitive routine diagnostics used in most if not all African countries. Nigeria with an estimated population of over 190 million people is rated amongst the highest TB burden countries with a total case notification of 104,904 of which 78% were bacteriologically confirmed pulmonary in 2017 alone [7]. The high prevalence of zoonotic TB in humans clearly follows the high prevalence of TB in cattle [8]. However, TB can move in both directions and studies have indicated isolation of *M. tuberculosis* from cattle and *M. bovis* from humans [9–12].

In most developing countries including Nigeria, TB diagnosis is mostly based on smear microscopy and only limited cultural isolation despite risk factors for zoonotic transmission of *Mycobacterium tuberculosis* complex (MTC) species. This situation is further worsened by lack of or limited health care facilities in most rural settings where pastoralists reside. In developed countries, however, the use of molecular epidemiological techniques like deletion typing, spoligotyping and Mycobacteria Interspersed Repetitive Units-Variable Tandem Repeat (MIRU-VNTR) has contributed significantly to revealing on-going transmission of disease between animals and humans [13] by providing precise epidemiological insights required for optimal prevention and control measures. This study was aimed at using molecular tools to establish zoonotic TB transmission link between pastoralists and cattle in Ebonyi State which has a TB prevalence of 3.2% by smear microscopy [14] and where the pastoralists live with their cattle in the same micro-environment.

## Results

### Culture and deletion analysis

From a total of 149 pastoralists screened for TB, 54 (36.2%) were positive for mycobacterial culture. Of these, deletion typing revealed 42 (77.8%) *M. tuberculosis*, one (1.9%) *M. bovis,* nine (16.7%) *M. africanum* and two (3.7%) nontuberculous mycobacteria (NTM) (Table 1) giving a TB prevalence of 34.9%. A total of 144 milk samples were obtained from 15 cattle herds in the state, out of which three animals (2.1%) were positive for mycobacterial culture. The deletion typing identified two (66.7%) of these as *M. tuberculosis* and the other (33.3%) as NTM (Table 1) giving an overall TB prevalence of 1.4% in the cattle studied.

**Table 1:** Distribution of *Mycobacterium tuberculosis* complex isolates according to sample types

Sample type	No. screened	No. culture positive (%)	Deletion typing No. (%)			
			*M. tuberculosis*	*M. bovis*	*M. africanum*	NTM
Milk	144	3 (2.1)	2 (66.7)	0 (0.0)	0 (0.0)	1 (33.3)
Sputum	149	54 (36.2)	42 (77.8)	1 (1.9)	9 (16.7)	2 (3.7)

### Spoligotyping and MIRU-VNTR

From the overall 54 MTC isolates obtained and subjected to spoligotyping (pastoralists =52, cattle =2), only 49 (pastoralists = 47, cattle = 2) showed clear and visible patterns. In all, 11 spoligotype patterns were obtained, resulting in an overall diversity (number of spoligotypes divided by the number of isolates, expressed as a percentage) of 22.5. A total of seven patterns occurred only once while four patterns were in clusters (*n* = 42) ranging from two to 29 isolates per cluster. Furthermore, classification based on family assignment revealed three previously known patterns including 59.2% Uganda I [SIT46- pastoralists: 28, cattle: 1], 16.3% Latin American Mediterranean- [SIT61, pastoralists: 8] and 2.0% T- [SIT53 – pastoralists: 1] strains of *M. tuberculosis*. Eleven MTC isolates yielded eight new strains [pastoralists: 10, cattle: 1] comprising one new strain each of *M. tuberculosis* and *M. bovis* (one isolate each) and six of *M. africanum* (nine isolates) (Table 2).

**Table 2:** Spoligotype patterns of MTC isolates from pastoralists and cattle in Ebonyi, Nigeria

Species/ spoligotype patterns	Number of isolates	No. in Cattle	No. in pastoralists	SIT*	Family
*M. bovis* (n = 1)	1	0	1		
	1	0	1	NEW	Af1
*M. tuberculosis* (*n* = 39)	39	2	37		
	8	0	8	61	LAM_CAM10
	29	1	28	46	Uganda I
	1	0	1	53	T
	1	1	0	NEW	Unique
*M. africanum* (*n* = 9)	9	0	9		
	1	0	1	NEW	Unique
	1	0	1	NEW	Unique
	1	0	1	NEW	Unique
	2	0	2	NEW	Unique
	3	0	3	NEW	Unique
	1	0	1	NEW	Unique
Total (*n* = 49)	49	2	47		

*SIT: Spoligo International Type

Nine of the ten selected isolates (pastoralists: 9, cattle: 1) from the predominant Uganda I family cluster commonly shared between the pastoralists and one of their cattle from the same microenvironment demonstrated genetic relatedness by showing same number of copies at each repetitive unit, indicative of established reverse zoonotic transmission of TB between pastoralists and cattle in the state (Table 3). One isolate from the pastoralists failed to amplify.

**Table 3:** Result of MIRU-VNTR typing of selected ten isolates from Uganda-I family cluster

	Isolate ID*									
	C1	P1	P 2	P 3	P 4	P 5	P 6	P 7	P 8	P 9
MIRU No.	No of repeats per locus									
MIRU 2	2 (508)**	2	2	2	2	2	2	2	2	Failed
MIRU 4	2 (276)	2	2	2	2	2	2	2	2	Failed
MIRU 10	6 (802)	6	6	6	6	6	6	6	6	Failed
MIRU 16	4 (777)	4	4	4	4	4	4	4	4	Failed
MIRU 20	3 (668)	3	3	3	3	3	3	3	3	Failed
MIRU 23	7 (518)	7	7	7	7	7	7	7	7	Failed
MIRU 24	2 (501)	2	2	2	2	2	2	2	2	Failed
MIRU 26	5 (540)	5	5	5	5	5	5	5	5	Failed
MIRU 27	4 (710)	4	4	4	4	4	4	4	4	Failed
MIRU 31	4 (704)	4	4	4	4	4	4	4	4	Failed
MIRU 39	3 (699)	3	3	3	3	3	3	3	3	Failed
MIRU 40	4 (570)	4	4	4	4	4	4	4	4	Failed
Mtub 04	3 (690)	3	3	3	3	3	3	3	3	Failed
ETR-C	3 (324)	3	3	3	3	3	3	3	3	Failed
Mtub 21	4 (320)	4	4	4	4	4	4	4	4	Failed
QUB-11b	4 (353)	4	4	4	4	4	4	4	4	Failed
ETR-A	3 (420)	3	3	3	3	3	3	3	3	Failed
Mtub 29	0 (335)	0	0	0	0	0	0	0	0	Failed
Mtub 30	3 (421)	3	3	3	3	3	3	3	3	Failed
ETR-B	3 (518)	3	3	3	3	3	3	3	3	Failed
Mtub 34	4 (542)	4	4	4	4	4	4	4	4	Failed
Mtub 39	4 (504)	4	4	4	4	4	4	4	4	Failed
QUB 26	4 (631)	4	4	4	4	4	4	4	4	Failed
QUB4156	3 (345)	3	3	3	3	3	3	3	3	Failed

*C – Cattle; P - Pastoralist **Base pair corresponding to the number of repeats

## Discussion

Available molecular epidemiological studies in Nigeria do not represent a confirmed picture of TB epi-links in the country despite the existing animal husbandry practices which enhance inter-transmission among humans and animals. Hence, TB control and informed prevention have been grossly limited. The present study reports a confirmed epi-link of reverse zoonotic TB transmission between pastoralists and cattle within the same microenvironment in Ebonyi State, Nigeria. To our knowledge, this is one of the first reports with confirmed TB inter-transmission amongst pastoralists and their cattle using 24 MIRU-VNTR in the country. Most previous reports on zoonotic transmission between humans and cattle in Nigeria were based on the speciation of the *Mycobacterium* without molecular confirmation of the genetic relatedness of the strains [15, 16] or conclusions drawn from infected humans and animals from mutually exclusive populations [10]. Indirect data suggesting that humans suffering from active TB are the most probable source of *M. tuberculosis* in cattle have been described before [17]. However, we consider this present finding to be one of the first unequivocal reports of human-to-cattle transmission of *M. tuberculosis* in Nigeria whereby we were able to confirm the genetic relatedness of the strain incriminated in the inter-transmission using molecular diagnostic techniques including 24 MIRU-VNTR.

Our findings showed that the reverse zoonotic transmission in the study area was due to an emerging Uganda I strain of *M. tuberculosis* from pastoralists to the infected cattle. The high prevalence of the Uganda I strain is an important epidemiological finding with significant implications for TB control. As observed, the Uganda I strain constituted 59.2% of the isolates recovered in the state. Generally, pastoralists are known for communal life-style, a critical factor that might promote human-to-human transmission of the strain [9]. Most of the pastoralists in the study area live within the same micro-environment and were known for congregational tendencies. These factors, coupled with poor practices (including unguarded close association between farmers and animals as well as consumption of unpasteurized milk) associated with this setting [9, 18] might further facilitate spread of infection from diseased to healthy individuals. Also, exacerbating risk factors such as HIV/AIDS, drug addiction, alcoholism and smoking are prevalent among pastoralists in developing countries, including Nigeria. Unfortunately, most of these workers are unaware of their health status, leading to considerable delays in seeking health care [9, 18]; thus promoting spread of infection from one person to the other. Besides, some modern strains are believed to exhibit more virulent phenotypes compared to ancient ones [19, 20], with emerging strains possessing inherent ability for adaptability and survival in the environment; thus circumventing routine treatment. This might further explain the reason for the high prevalence of this emerging strain.

The isolation of the two *M. tuberculosis* from milk samples in this study could not be as a result of contamination of milk by cough spray from infected humans. This is because the milk samples were collected from individual animals in an aseptic manner into sterile containers, showing the excretion of this organism from the cattle through milk. This is in line with previous reports of *M. tuberculosis* isolation in cow milk from several parts of the world [21–23]. As previously reported, humans are the initial source of *M. tuberculosis* infection for animals [22], with potential for this infection being carried back to humans. Vordermeier et al. [17] in a study in Ethiopia reported that though the extent and risk of infections caused by *M. bovis* are unclear, the facts that *M. tuberculosis* could be isolated from tuberculous cattle is a demonstration of a potential cattle-to-human transmission risk. The impact of this disease can be devastating in poor or limited resource countries characterized with high burdens of both TB and HIV [24]. Therefore, control of TB due to *M. tuberculosis* in humans does not depend only on the control of transmission among humans but also include breaking the transmission route particularly through bovine milk.

From this study, a new strain of *M. bovis* was detected in a pastoralist showing a possible zoonotic transmission from infected cattle. A previous study reported evidence of occupational exposure through the isolation of *M. bovis* from livestock workers in Ibadan, Nigeria [9]. Other studies have also confirmed *M. bovis* in humans including from Tanzania [25], Zaire and Egypt [5, 26]. In addition, the *M. bovis* isolates obtained in this study belonged to Af1 strains given their lack of spacer 30 which are known to be predominant in Cameroon, Nigeria and Mali [27].

The study also reported isolation of *M. africanum* from the pastoralists. This finding is in line with previous report that indicated that *M. africanum* strains are known as causative agents of human tuberculosis in West Africa [28]. Besides, evidence of person to person transmission of *M. africanum* has also been reported [29], thus suggesting that an animal reservoir is not fully required.

This study, however, has some limitations. Firstly, the full clinical details of the infected pastoralists were not obtained. This could have given further credence to the findings observed from this study. Secondly, only cows were screened, whereas bulls could also harbour the infection. However, screening bulls would require tuberculin testing followed by slaughter and post-mortem of positive reactors. This would be problematic, since most farmers do not sell their animals except when in dire need of cash or the animals are unproductive.

## Conclusions

The study provides a first report of a confirmed epi-link of reverse zoonotic TB transmission between pastoralists and cattle in Nigeria using molecular diagnostic techniques as 24-MIRU-VNTR. It established that the epi-link was due to an emerging *M. tuberculosis* Uganda I strain circulating between pastoralists and cattle in Ebonyi State, Nigeria. We also report that the high proportions of this emerging strain has implications for TB epidemiology and control. Isolation of *M. tuberculosis* from cattle and *M. bovis* from the pastoralist further reiterates the zoonotic nature of TB. Hence, clinical laboratories should routinely use molecular tests to identify mycobacterial species and differentiate *M. bovis* from *M. tuberculosis*. Finally, it shows that detection of zoonoses with the aim of informed disease control in animals and humans could be enhanced through molecular-guided cooperation between human and veterinary health services.

## Methods

### Study site and setting

This cross-sectional study carried out in Ebonyi State, south-eastern Nigeria was a step further to our initial study which focused on determining genetic diversity of *Mycobacterium tuberculosis* complex strains in livestock workers and cattle in Nigeria [15]. The present study was aimed at using molecular tools including 24 MIRU-VNTR to establish zoonotic TB transmission link between pastoralists and cattle in the state. The majority of pastoralists in the state known for communal life-styles, live in farm settlements within the same vicinity with regular opportunities for convergence through social gatherings and cultural rites. This thus creates an environment conducive for intra- and inter-transmission of TB from infected to non-infected cattle and humans.

### Sample size and sample collection

#### Human sampling

Cattle farm settlements were identified through the Ministry of Agriculture and Natural Resources in the state. Following visits paid to the traditional head of the settlements, a meeting was convened with all potential participants where the objectives and benefits of the study were explained to them. Oral consents were thereafter obtained from those willing to participate given the low educational status of most o f the pastoralists. Based on 7.1% prevalence of *M. bovis* infection among livestock workers in Nigeria [30], an estimated minimum sample size of 101 participants was obtained following the procedure for sample size estimation [31]. However, a total of randomly selected 149 pastoralists participated in the study including their wives, children who were at least 18 years old as well as their cattle handlers. Sputum samples were consecutively collected early in the morning over a period of six months using sterile, plastic sample containers from one of every four household members who consented to participate in the study until the sample size was reached. They were guided to void the sputum samples in open air and not in confined or overcrowded places in order to avoid generating infective aerosols. Identification code was written on each sputum container and all collected samples were placed in an ice-packed cool flask.

#### Animal sampling

All lactating cows from the herds of the consenting participants were selected, from which 10 mL of milk was aseptically collected into a 20 mL sterile sample container through the assistance of the livestock owners. The udders of the individual cows were cleaned and disinfected before the collection of the sample in order to avoid contamination of the milk sample. The collected samples were appropriately labelled and kept in a cool flask containing ice-packs. Data on the size of the herds was documented.

All samples collected from the pastoralists and their cattle were transported in the respective cool transport flasks to the Tuberculosis Laboratory of the Department of Veterinary Public Health and Preventive Medicine, University of Ibadan, where they were refrigerated prior to processing. Each batch of samples were processed within one-week post-collection.

#### Sample processing

Milk sample processing was done on separate days apart from sputum samples to eliminate risk of cross-contamination. Samples were processed following a described procedure using Mycoprep decontaminant [32]. The concentrates thus obtained each from sputum and milk samples were inoculated onto two slopes of Löwenstein-Jensen with either glycerol or pyruvate and incubated at 37 °C for 12 weeks. Positive growths were harvested and subjected to deletion typing to identify *M. tuberculosis* complex isolates by the polymerase chain reaction (PCR) amplification of genomic regions of difference (RD), as described elsewhere [33]. Isolates identified as members of MTC were thereafter processed by spoligotyping as earlier reported [15] following a standardized international method previously described [34] using a commercially available kit (Isogen Biosciences BV, Maarsen, The Netherlands). Briefly, 25 μl of amplified PCR product was diluted in 160 μl of 2X SSPE–0.1% SDS and denatured by heat at 72 °C for 10mins. The diluted samples were immediately placed on ice and then 150 μl of each diluted sample was pipetted into respective parallel channel of the membrane in such a way that the channels of the miniblotter apparatus were perpendicular to the rows of oligonucleotides on the membrane. Hybridization was done for 60 mins at 60 °C without allowing shaking of the miniblotter to avoid cross contamination of samples. After hybridization, the samples were removed from the membrane using aspirator. The membrane was then washed twice in 250 ml of 2X SSPE/0.5% SDS for 10 min each time at 60 °C and then incubated in diluted streptavidin-peroxidase conjugate (Boehringer) and 2X SSPE/0.5% SDS at 1:4000 for 60 min at 42 °C. The membrane was washed twice, for 10 min each time, in 250 ml of 2X SSPE/0.5% SDS at 42 °C and rinsed with 250 ml of 2X SSPE for 5 min at room temperature. Detection of hybridizing DNA was done by incubating the membrane in 20 mL chemiluminescent ECL (Amersham) detection liquid for 1.5mins, followed by exposure to X-ray film (Hyperfilm ECL; Amersham) for 20 min in accordance with the instructions of the manufacturer. *Mycobacterium tuberculosis* H37Rv, *M. bovis* Bacille Calmette-Guérin and sterile distilled water were used as controls. Resulting spoligotype patterns were compared to existing patterns in an international spoligotyping database (SITVIT2) [35] available at http://www.pasteur-guadeloupe.fr:8081/SITVITDemo/. Spoligotype patterns were grouped as spoligotype international types (SITs) if they shared identical spoligotype patterns with patterns present in the existing database. Spoligotype families were assigned as previously described [35]. Of these spoligotype families identified and earlier published [15], selected isolates from the predominant cluster commonly shared between pastoralists and cattle belonging to the same microenvironment were genotyped by PCR amplification of 24 MIRU-VNTR as earlier described [36] using primers that amplify 24 polymorphic loci on the mycobacterial genome per DNA isolate. Briefly, 2 μl of mycobacterial DNA was added to a final volume of 25 μl containing 8.375 μl of free RNase water (Qiagen, USA), 5 μl of Q solution, 2.5 μl of 10*x* buffer, 2 μl of 1.5 mM MgCl_2_ (Roche, USA), 4 μl of 0.2 mM dNTPs (Promega, WI USA), 1 μl primer and 0.125 μl of HotStar Taq polymerase (1 U). The PCR conditions included three stages: initial denaturation at 95 °C for 15 min (Stage 1), second denaturation at 94 °C for 1 min, annealing at 62 °C for 1 min, initial extension for 1 min at 72 °C (Stage 2) and final extension at 72 °C for 10 min followed by cooling to 4 °C prior to analysis (Stage 3). A 45-cycles PCR was done on Veriti™ 96-well Thermal Cycler (Applied Bio system, Singapore). The laboratory *M. tuberculosis* H37Rv reference strain DNA was used as positive control and DNA-free water as a negative control. Amplification products were electrophoretically fractionated in 1% agarose gel (SeaKem® LE) in 1*x* TE buffer at 160 V for 4 h to allow maximum fragment size separation for clear discrimination. The number of tandem repeat units present at each locus was calculated from the size of DNA fragments according to a standardized table (http://www.MIRU-VNTRplus.org). The results were expressed in digital format where each number represented the number of repeat copies at a particular locus. The isolates from the pastoralists were those from cattle handlers belonging to the same herd with the cattle positive for the shared cluster.

## Data Availability

The datasets used and/or analysed during the current study are available from the corresponding author on reasonable request.

## Abbreviations

ACUREC: Animal care, use and research ethics committee
BTB: Bovine tuberculosis
DNA: Deoxyribonucleic acid
ECL: Electrochemiluminescence
HIV/AIDS: Human infectious virus/acquired immunodeficiency syndrome
MIRU-VNTR: Mycobacterial Interspersed Repetitive Unit-Variable Number Tandem Repeats
MTC: *Mycobacterium tuberculosis* complex
NTM: Nontuberculous mycobacteria
PCR: Polymerase chain reaction
RD: Regions of difference
SDS: Sodium dodecyl sulfate
SIT: Spoligo international type
SSPE: Saline-sodium phosphate EDTA
TB: Tuberculosis
